# Evaluating the Predictivity of Virtual Screening for Abl Kinase Inhibitors to Hinder Drug Resistance

**DOI:** 10.1111/cbdd.12170

**Published:** 2013-10-01

**Authors:** Osman A B S M Gani, Dilip Narayanan, Richard A Engh

**Affiliations:** 1 The Norwegian Structural Biology Center, Department of Chemistry, University of Tromsø9037, Tromsø, Norway

**Keywords:** cheminformatics, docking, kinase, virtual screening

## Abstract

Virtual screening methods are now widely used in early stages of drug discovery, aiming to rank potential inhibitors. However, any practical ligand set (of active or inactive compounds) chosen for deriving new virtual screening approaches cannot fully represent all relevant chemical space for potential new compounds. In this study, we have taken a retrospective approach to evaluate virtual screening methods for the leukemia target kinase ABL1 and its drug-resistant mutant ABL1-T315I. ‘Dual active’ inhibitors against both targets were grouped together with inactive ligands chosen from different decoy sets and tested with virtual screening approaches with and without explicit use of target structures (docking). We show how various scoring functions and choice of inactive ligand sets influence overall and early enrichment of the libraries. Although ligand-based methods, for example principal component analyses of chemical properties, can distinguish some decoy sets from active compounds, the addition of target structural information via docking improves enrichment, and explicit consideration of multiple target conformations (i.e. types I and II) achieves best enrichment of active versus inactive ligands, even without assuming knowledge of the binding mode. We believe that this study can be extended to other therapeutically important kinases in prospective virtual screening studies.

The availability of crystal structures of many key drug targets and the low cost of computational methods now encourage the use of virtual screening (VS) in early stages of drug discovery. There is an enormous quantity of data regarding target structures and ligand binding, and VS should be expected to work best when all experimental knowledge is integrated appropriately into the methods. If the ligand set contains diverse or focussed scaffolds, then the training or parameterization of the VS method should be designed to account for this. Screening of focussed databases will best predict active ligands when trained against similar compounds, and screening of diverse sets will best identify active ligands if the variability of the target protein is adequately represented in the method. In this study, we examine VS approaches for the leukemia target receptor ABL1, a protein tyrosine kinase now well characterized by knowledge of multiple inhibitors and target conformations.

Inhibition of protein kinases by selective inhibitors has become a major therapeutic approach for many diseases, especially well established for cancer. Targeted inhibition of ABL1 and several related kinases by imatinib (Gleevec, Novartis) has become the successful front-line therapy for chronic myeloid leukemia (CML) and several solid tumors 1. Response to imatinib therapy in CML statistically is highly durable in the chronic phase; especially with early initiation of treatment; more advanced stages of the disease often involve relapse and imatinib resistance 2,3. Mutations of amino acids in the kinase domain of ABL1 are the most common cause of such resistance, affecting some 50–90% patients with acquired resistance 4–6. Among the various mutations, an isoleucine substitution at the ‘gatekeeper’ residue threonine (T315I) accounts for about 20% of the total burden of clinical resistance 5. This residue has been designated ‘gatekeeper’ due to its position that determines the size of a hydrophobic pocket in the active site of the kinase domain. Many small molecule inhibitors exploit this threonine residue for their specificity 7. Substitution of the gatekeeper residue has been observed as a major mechanism of acquired resistance for other tyrosine kinase drug targets, including c-KIT-T670I 8, EGFR-T790I 9, and PDGFRalpha-T74M/I 10.

Recent studies have shown a strong correlation between substitution of the gatekeeper residue and oncogenic transformation 11, and substitution of a threonine gatekeeper residue with a hydrophobic residue such as leucine is a mechanism of activation of several tyrosine kinases 12. Thus, the mechanism of resistance against Abl inhibitor drugs involves not only drug binding properties, but also the oncogenic transformation capacity of gatekeeper mutant itself. Second-generation CML drugs, such as dasatinib and nilotinib, have been introduced to combat or forestall resistant forms. However, many of these newer drugs do not eliminate resistance via the gatekeeper mutation (ABL1-T315I) 4,13, despite greater potency against wild-type protein (ABL1-wt) and most of the imatinib-resistant mutations 13–15. Therefore, developing ABL1 inhibitors that target resistance mutations, and in particular the ABL1-T315I gatekeeper mutation, currently remains a goal of leukemia drug research.

Known inhibitors of ABL1 that also inhibit the ABL1-T315I form are predominantly ‘type II’ inhibitors, targeting an inactive conformation of the kinase. These include ponatinib (in clinical trials, also known as AP2453416, along with others in earlier stages of development) 16,17. Type II inhibitors bind in a deep and mostly hydrophobic pocket that exists when the activation loop of a kinase adopts an inactive conformation in which the phenylalanine of the conserved DFG motif is removed from its hydrophobic packing position that becomes the pocket. Other characteristics of type II inhibitors include hydrogen bonding interactions, usually involving amide or urea moieties. In contrast, type I inhibitors bind to the active form of the kinase, in which the DFG phenylalanine is bound in its hydrophobic site, and the neighboring aspartate is positioned appropriately for its role in the phosphotransfer reaction of the kinase. Both type I and type II inhibitors typically bind to the hinge region that also anchors the ATP adenine via hydrogen bonds. Figure1 shows type I and type II binding conformations of ABL1 kinase domain structures.

**Figure 1:**
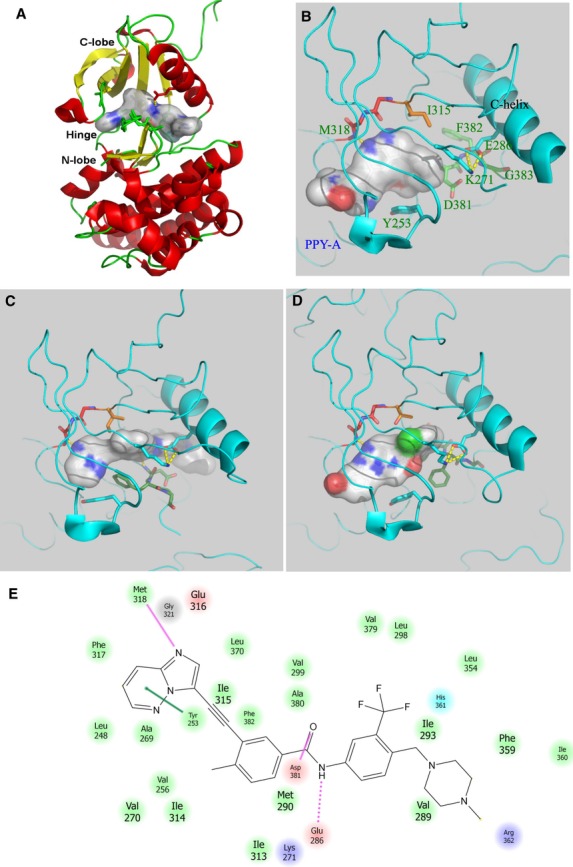
Representative active and inactive conformations of the ABL1 kinase domain. (A) Overall kinase domain structure of ABL1. The major structural features (C-lobe, N-lobe, and hinge) are labeled. The ligand (ponatinib) is represented by a stick model surrounded by a solvent accessible surface. (B) The active DFG-in conformation, target form for type I inhibitors, is shown here taken from Protein Databank (PDB) entry 2z60 with inhibitor PPY-A. The phenylalanine of the DFG motif is packed into its hydrophobic spine position, and the DFG aspartic acid is in a position able to coordinate Mg ions for ATP binding. (C) The DFG-out configuration is shown here for type II inhibitor ponatinib (3ik3). The DFG phenylalanine is removed from its active position, and the activation loop is greatly displaced. (D) An inactive conformation of ABL1 bound to inhibitor PD166326 (1opk) is intermediate between ‘DFG-in’ and ‘DFG-out’. The DFG phenylalanine is removed from its active position, but the overall activation loop main chain resembles an active conformation. The salt bridge between the conserved glutamic acid emerging from the C helix and the catalytic lysine residue from beta strand 3 is present. (E) Overview of ABL1 interactions with type II inhibitor ponatinib.

We studied a set of high-potency ABL1 inhibitors that can inhibit both ABL1-wt and ABL1-T315I forms (Figure2). Applying VS retrospectively to these and related inhibitors, we aimed to identify VS protocols that best identify active inhibitors dispersed in larger libraries. The protocols vary with respect to the chemical properties analyzed, and the amount and type of target structural information integrated into the procedures. Such optimized protocols would be best suited to screen libraries of ligands with unknown activity against ABL1 and mutant forms. The study can in principle be extended to other therapeutically important kinases and also provides information for the extent of structural information needed for success.

**Figure 2:**
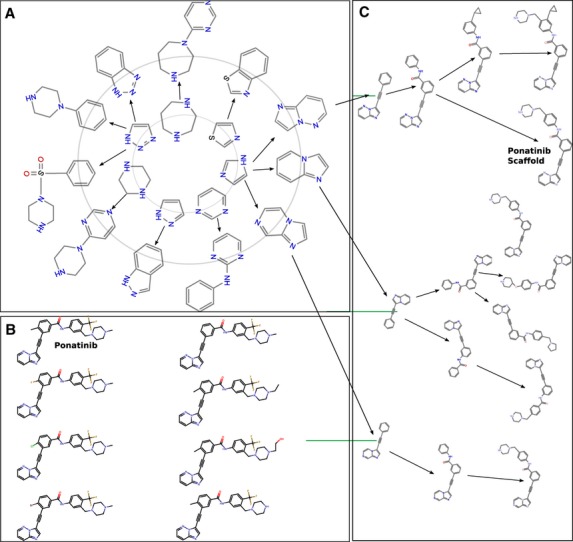
Scaffold tree of high-affinity dual inhibitors for ABL1-wt and ABL1-T315I. Imidazole is the parent scaffold that gives rise to all ponatinib analogs. (A) First two parent layers of the scaffold tree. (B) Full extension of the imidazole containing scaffolds: the ponatinib containing scaffold is marked. (C) All inhibitors derived from ponatinib scaffold. The term ‘analog’ is used loosely in this article. The inhibitors that are visually similar to ponatinib in 2D sketches are termed analogs. Scaffold is a well-defined term in this article. A scaffold consists of all carbo- and heterocyclic rings, their aliphatic linker bonds, and atoms attached via a double bond. Therefore, the inhibitors that have similar structures but differ in heterocyclic atoms are not considered to have the same parent scaffold.

## Methods and Materials

### ABL1 inhibitor set

To create a library of inhibitors that inhibit both ABL1-wt and ABL1-T315I, representing a set of active compounds with decreased drug resistance potential, compounds with IC_50_ values <100 nm in enzyme assays for ABL1-wt or ABL1-T315I were retrieved from the Kinase Knowledgebase (KKB, www.eidogen-sertanty.com). Of the inhibitors identified, 38 were inhibitory (IC_50_ < 100 nm) for both the wild-type and mutant forms; 16 of these were ponatinib analogs. In addition, 141 were inhibitory for ABL1-wt alone (IC_50_ for ABL1-T315 > 1 μm or no mutant binding data available). In contrast, all the high-potency inhibitors of ABL1-T315I were active against the wild-type target (IC_50_ < 1 μm). Here, we study the dual high-potency (IC_50_ < 100 nm) inhibitors in detail, as they possess in common one of the selectivity criteria for ABL inhibition therapy that aims to reduce the occurrence of drug resistance. Table1 summarizes the sizes of the relevant inhibitor sets taken from the KKB database.

**Table 1:** ABL1 inhibitors existing in kinase knowledgebase (KKB). An inhibitor can be counted for both wild-type and mutant forms

IC_50_ (nm)	ABL1-wt	ABL1-T315I
<100	232	60
100–299	68	79
300–1000	48	60

**Figure 3:**
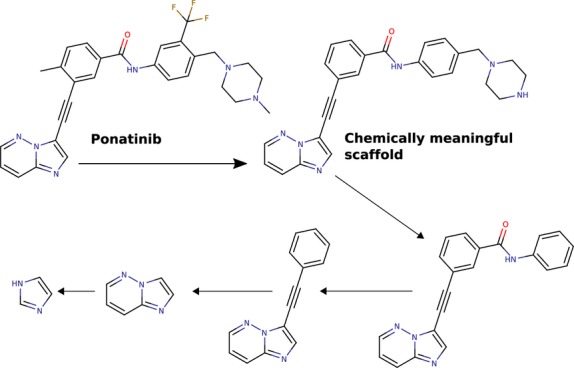
Scaffold generation process. Taking ponatinib as an example, a chemically meaningful scaffold is extracted and successively deconstructed one ring at a time.

The diversity of this inhibitor set was analyzed by the Scaffold Hunter program 18. A scaffold is defined by the all carbon and heterocyclic rings, their aliphatic linker bonds, and atoms attached via a double bond 19. Scaffold Hunter extracts chemically meaningful compound scaffolds and iteratively removes one ring at a time to generate smaller compounds. Thereby, a hierarchical arrangement of parents and children is formed, yielding branches that are combined to form a tree (Figure3).

### Inactive ligand sets

Three ‘decoy’ sets were chosen for inclusion into test libraries that combine active and inactive compounds. The largest set was retrieved from the Directory of Useful Decoys (DUD) 20, containing 6319 physically similar but topologically distinct ligands. As no decoy set chosen explicitly for ABL kinase domains is available from DUD, the decoy set for homologous kinase SRC was used for this study. A second set was taken from Glide 21. This set is ‘universal’, that is, neither ‘kinase inhibitor-like’ nor specifically ‘non-kinase-inhibitory’, consists of 1000 ligands and was created from one million druglike ligands. Finally, a set was chosen from the weak binding inhibitors (enzyme inhibition IC_50_ = 100–1000 nm), containing 89 inhibitors. As weak binders, these might be considered the most challenging decoys.

### ABL1 kinase domain structures

Five crystal structures of T315I mutants of ABL1 kinase domain in complex with inhibitors were taken for analysis, along with structures for four of these inhibitors that have been co-crystallized also with the ABL1-wt kinase domain. These structures, summarized in Table2, were used for VS of dual active inhibitors and of inactive ligands. Because four pairs of structures, each with one inhibitor binding both the wt and T315I forms, are included, the test set includes a range of inhibitor-associated flexibilities, DFG conformational states, and allows direct comparisons of the effects of gatekeeper mutations.

**Table 2:** ABL1 kinase domain structures deposited in the Protein Databank (PDB). IC_50_ values of the co-crystallized inhibitors and some structural features are also listed. The X-ray crystallographic resolution is shown in braces

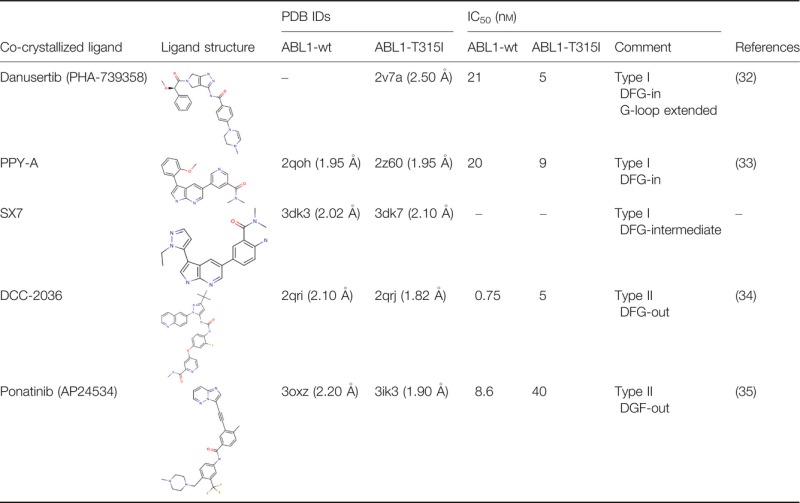

### Virtual screening studies

#### Protein preparation

For docking, the single kinase domain structures, in complex with their native ligands, were analyzed by the protein preparation wizard of Schrodinger program (Schrodinger LLC, 2011, New York, NY, USA). Water molecules were deleted, bond orders assigned, and hydrogen atoms were added. A restrained minimization was then performed with the OPLS2005 force field using the default constraint of 0.30 Å RMSD. A grid box was then generated for each structure that included co-crystallized ligand and most of the binding cleft between the N- and C-lobes. The main chain nitrogen of Met318 at the hinge segment of kinase domain was included as constraint as a hydrogen bond donor for the docking runs.

#### Ligand preparation

Ligand preparation and the subsequent calculations were performed by modified KNIME (www.knime.org) workflows made up of Schrodinger modules. The co-crystallized ligands, the dual active inhibitors, and decoy sets mentioned in the ligand-based study were prepared using the OPLS2005 force field in the ligand preparation module of Schrodinger. The ligands were ionized as between pH 5–9, and the tautomers and stereoisomers were generated. Finally one lowest energy conformation from the generated conformer set was chosen for docking with Glide.

#### Docking and scoring protocol

The compounds of the libraries were classified into ‘hits’ – a ranked list – and ‘inactives’ using three different Glide docking protocols: high throughput virtual screening (HTVS), standard precision (SP), and extra precision (XP). For each ligand, Glide generates a set of low-energy conformations and then exhaustively searches the receptor active site to position the conformers. The docked poses pass through a series of hierarchical filters that evaluate the receptor–ligand interactions and are then energy-minimized on a precomputed grid of van der Waals and electrostatic energies for the receptor. The final scores are calculated according to the energy functions described elsewhere 22. In short, all docking functions use flexible ligand docking and same scoring scheme. But HTVS reduces the number of low-energy conformers through the docking filters. Moreover, HTVS reduces the thoroughness of the final torsional refinement and sampling of the ligand conformers. Compared with XP, SP is a softer method that can identify relatively weak binders by allowing ‘less than perfect’ poses. Therefore, SP is used in large-scale VS to identify ligands with a reasonable propensity to bind. Extra precision imposes severe penalties for poses that apparently violate physical chemistry rules. For example, charged and strongly polar groups should be adequately exposed to solvent. Extra precision thereby reduces false positives and can be used in lead optimization studies where only a limited number of compounds are considered for synthesis or other experiments.

#### MM-GBSA re-scoring

To estimate the free energy of binding between the receptor and the ligands, an implicit solvation model was used via the molecular mechanics – generalized Born surface (MM-GBSA) approach. Glide SP poses were re-scored using MM-GBSA in two ways: first, as a rigid receptor, and secondly, as a partially flexible receptor where any residue with an atom within 12 Å of the ligand remained flexible.

The MM-GBSA is a postprocessing end-state method for calculating free energies of binding of molecules in solution. Compared with more rigorous methods such as free energy perturbation and thermodynamic integration methods, MM-GBSA and the related method MM-PBSA are computationally more efficient. All these methods allow for rigorous free energy decomposition into contributions from different groups of atoms or types of interaction. In MM-GBSA, the binding free energy (ΔG_bind)_ between a ligand (L) and a receptor (R) in forming the complex (RL) is calculated as: 

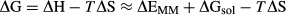
1

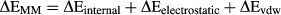
2

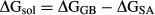
3where ΔE_MM_, ΔG_sol_ and ΔS denote the change in gas phase MM energy, solvation free energy, and the conformational entropy upon binding. ΔE_MM_ is composed of ΔE_internal_ (bond, angle, and dihedral energies), ΔE_electrostatic_, and ΔE_vdw_ (van der Waals) energies. ΔG_sol_ is the sum of electrostatic solvation energy (polar contribution), ΔG_GB_, and the non-electrostatic solvation component (non-polar contribution), ΔG_SA_. The polar contribution is calculated using either the GB or PB model, while the non-polar energy is estimated by solvent accessible surface area.

In Schrodinger, the calculation is performed in following steps:

1. Minimization of receptor alone
2. Minimization of ligand alone
3. Energy calculation after ligand extraction from optimized receptor-ligand complex
4. Energy calculation after receptor extraction from optimized receptor-ligand complex

#### Docking analyses

Two metrics were used to calculate the enrichment success of the virtual screening output ‘hit’ lists: the enrichment factor (EF) and the receiver operating characteristic (ROC) plot. The EF plots the percentage of actives as a function of the position in the ranked list versus percentage of all hits from the database. Active ligands or decoys were identified as hits once they pass the Glide docking filters mentioned above and can be ranked according to Glide docking scores. In an XY plot for EF calculation, 

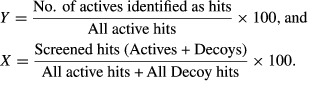


The EF was calculated for 1%, 5%, and 10% of the total hits that contain active ligands and decoys. This method approximates and tests reasonable procedures of selecting compounds for testing after ranking compounds of unknown activity by VS.

Receiver operating characteristic plots true positive rates in *Y*-axis and the corresponding true positive rate in *X*-axis: 

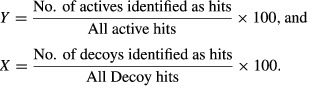


The area under the curve (AUC) of ROC plot is equivalent to the probability that a VS run will rank a randomly chosen active ligand over a randomly chosen decoy.

The EF and ROC methods plot identical values on the *Y*-axis, but at different *X*-axis positions. Because the EF method plots the successful prediction rate versus total number of compounds, the curve shape depends on the relative proportions of the active and decoy sets. This sensitivity is reduced in ROC plot, which considers explicitly the false positive rate. However, with a sufficiently large decoy set, the EF and ROC plots should be similar.

### Ligand-only-based methods

In principle, (ignoring the practical need to restrict chemical space to tractable dimensions), given enough data on a large and diverse enough library, examination of the chemical properties of compounds, along with the target binding properties, should be sufficient to train cheminformatics methods to predict new binders and indeed to map the target binding site(s) and binding mode(s). In practice, such SAR approaches are limited to interpolation within structural classes and single binding modes, partly because of the amount of data available and also partly because of the consequently limited number of chemical descriptors considered. Here, in order to investigate to what extent the active inhibitors and decoys can be distinguished, the compounds were assigned chemical space coordinates according to the molecular descriptor-based principal component (PC) sets of ChemGPS-NPweb 23. These descriptors include some 40 molecular descriptors such as molecular weight, number of rotatable bonds, number of hydrogen bond donors/acceptors and were analyzed for active ligands, DUD decoys, and randomly selected high-potency (IC_50_ < 100 nm) kinase inhibitors. The first three PCs from the ChemGPS-NPweb-based calculations can distinguish the inhibitor and decoy compound sets (with some overlap), but the ABL1 inhibitors are found scattered and indistinguishable within the volume populated by randomly chosen kinase inhibitors (IC_50_ < 100 nm). The first four dimensions of the ChemGPS-NP PC calculation account for 77% of the data variance. For typical compound sets, PC1 represents size, shape, and polarizability; PC2 corresponds to aromatics and conjugation-related properties; PC3 describes lipophilicity, polarity, and H-bond capacity; and PC4 expresses flexibility and rigidity. A 3D plot was constructed from the three-first PCs to display the distinctions between the various compound sets.

Correlation of molecular properties and binding affinity: The Canvas module of the Schrodinger suit of programs provides a range of methods for building a model that can be used to predict molecular properties. They include the common regression models, such as multiple linear regression, partial least-squares regression, and neural network model.

Several molecular descriptors and binary fingerprints were calculated, also using the Canvas module of the Schrodinger program suite. From this, models were generated to test their ability to predict the experimentally derived binding energies (pIC_50_) of the inhibitors from the chemical descriptors without knowledge of target structure. The training and test set were assigned randomly for model building.

### Neural network regression

Neural networks are biologically inspired computational methods that simulate models of brain information processing. Patterns (e.g. sets of chemical descriptors) are linked to categories of recognition (e.g. binder/non-binder) via ‘hidden’ layers of functionality that pass on signals to the next layer when certain conditions are met. Training cycles, whereby both categories and data patterns are simultaneously given, parameterize these intervening layers. The network then recognizes the patterns seen during training and retains the ability to generalize and recognize similar, but non-identical patterns.

## Results

### Diversity of the inhibitor set

**Figure 4:**
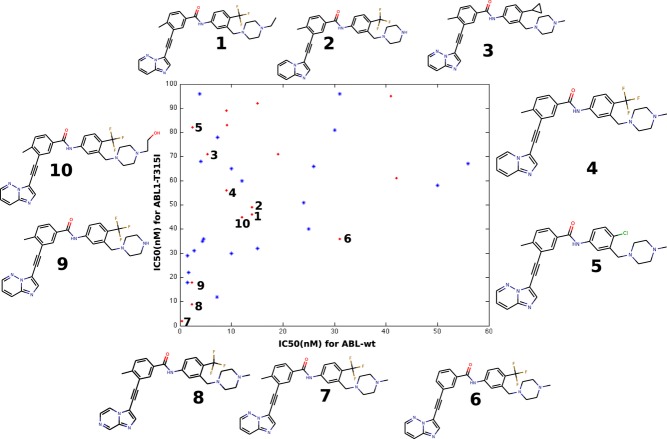
Scatter plot of high-affinity inhibitors of wild-type and T315I mutant ABL1. Selected ponatinib analogs show how ABL1-T315I inhibition varies among close analogs.

The high-affinity dual inhibitors for wt and T315I ABL1 kinase domains can be divided roughly into two major scaffold categories: ponatinib-like and non-ponatinib inhibitors. The scaffold analysis shows that there are some 23 major scaffolds in these high-affinity inhibitors. Although ponatinib analogs comprise 16 of the 38 inhibitors, they are constructed from seven child scaffolds (Figure2). These seven child scaffolds give rise to eight inhibitors, including ponatinib. However, these closely related inhibitors vary significantly in their binding affinity for the T315I isoform of ABL1, while wt inhibition values are similar (Figure4).

Figure4 shows clearly that T315I affinity for ponatinib analogs vary according to variations in their hydrophobic binding interactions. For example, replacement of CF_3_ by a chlorine atom causes a dramatic decrease in affinity for T315I. A similar effect can be observed for 4-methyl substitution at the piperazine ring. Thus, the ponatinib scaffold provides the greatest binding energy components via predominantly polar interactions, especially H-bonding at the hinge, but variations in the side chains and their mostly hydrophobic interactions cause the variations in binding affinity seen mostly for binding to the T315I isoform.

#### HTVS and SP docking with DUD decoys

**Figure 5:**
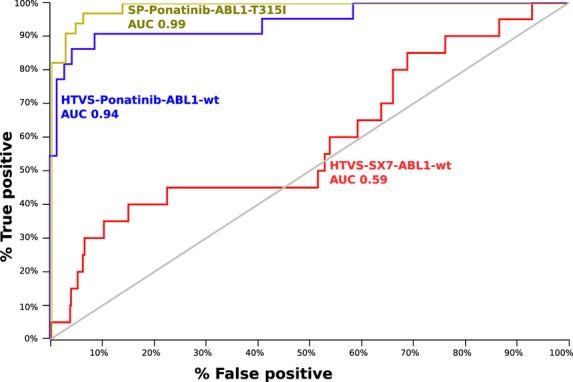
Receiver operating characteristic (ROC) plots of the selected docking runs. The light gray diagonal line shows hypothetical random performance, with an area under the curve (AUC) of 0.50. The overall and early enrichment are low with type I ABL1 conformation as target using the high throughput virtual screening (HTVS) method. With type II conformations, enrichments are better, especially for the standard precision (SP) method (compared with HTVS).

Virtual screening docking runs were performed for the library of dual active compounds dispersed in the DUD decoy set against the nine ABL1 kinase domains as summarized in Table2. For each kinase domain target structure, the co-crystallized ligand, the dual active inhibitors, and the DUD sets were docked using the HTVS and SP modes. The resulting ranked hit lists were characterized using the EF and ROC AUC methods (Table3, Figure5). The AUC values show that – with a single exception – SP docking shows better results compared with the HTVS protocol (Table3). The exception occurs for docking against the PPY-A-bound ABL1-T315I structure. Docking to the type II receptor conformations in general provided much higher enrichment of active inhibitors. Nearly 99% enrichment was obtained by docking against each of the type II conformation structures of ABL1-T315I. For VS against a single target structure, the ROC AUC values from the SP docking highlight the type II ABL1-T315I kinase domain structure as the best choice.

**Table 3:** Docking of high-affinity inhibitors onto ABL1 kinase domains. The results are shown as ROC AUC values

Type	Ligand of target kinase	ABL1-wt		ABL1-T315I	
		HTVS	SP	HTVS	SP
Type I	Danusertib	–	–	0.70	0.74
	PPY-A	0.77	0.78	0.90	0.82
	SX7	0.59	0.88	0.69	0.93
Type II	DCC-2036	0.86	0.97	0.88	0.99
	Ponatinib	0.87	0.96	0.94	0.99

AUC, area under the curve; HTVS, high throughput virtual screening; ROC, receiver operating characteristic; SP, standard precision.

#### Evaluation of early enrichment factors

The early EFs calculated for the VS runs are shown for the SP method in Table4, highlighting the relative success of the docking runs to identify actives, filter away decoys, and rank actives over the remaining decoys in the hit list. Both the type II conformation targets provide the best results. As the best example, docking against the ponatinib-bound ABL1-T315I kinase domain structure, 34 (89%) of 38 active inhibitors versus only 1915 (30%) of 6319 decoys were identified as hits. At the EF1% level, 18 (47%) of these active inhibitors were already included. The superior performance of the type II conformation target structures is perhaps not surprising, given the preponderance of type II inhibitors in the dual active set. However, there are significant differences between the docking runs against the two type II target structures. Against the DCC-2036 bound kinase domains, enrichment of the active inhibitors was a bit higher, but at the cost of identifying more than 70% of decoys as hits. However, some of the discouragement of this result is compensated for by the relatively high early enrichment values. Using type I kinase domain conformations, more actives and decoys were identified as hits – up to 80% of the decoys – and early enrichments were much poorer than using the type II conformation as docking target.

**Table 4:** Overall and early enrichment of high-affinity inhibitors in SP docking. All values are shown in percentage

Ligand of target kinase	Actives identified as hits		Decoys identified as hits		EF1%		EF5%		EF10%	
	ABL1-wt	ABL1-T315I	ABL1-wt	ABL1-T315I	ABL1-wt	ABL1-T315I	ABL1-wt	ABL1-T315I	ABL1-wt	ABL1-T315I
Danusertib	–	100		79	–	21	–	50	–	61
PPY-A	100	100	80	80	37	37	39	47	53	61
SX7	100	100	80	80	11	26	58	68	74	84
DCC-2036	97	95	70	51	65	61	86	86	92	97
Ponatinib	95	89	55	30	67	47	86	82	94	87

EF, enrichment factor; SP, standard precision.

#### Binding energy prediction and enrichment with MM-GBSA

Binding energies were calculated for the SP docked poses using MM-GBSA, which in theory should provide improved energy values and, by extension, should improve the ranking of the hit list. However, Table5 shows that both the ROC AUC and enrichment values are decreased for type II conformation targets with MM-GBSA approach. For the type I, the results were mixed. Although the overall enrichments were generally increased compared with the SP and HTVS approaches, the early enrichment values are lowered in most cases. These values show that binding energies calculated by MM-GBSA approach could enrich the active inhibitors from decoys, but the performance was less satisfactory than SP docking energies.

**Table 5:** ROC AUC and early enrichments by MM-GBSA energies on SP docked poses

Ligand of target kinase	ABL1-wt				ABL1-T315I			
	ROC AUC	EF1%	EF5%	EF10%	ROC AUC	EF1%	EF5%	EF10%
Danusertib	–	–	–	–	0.82	13	55	63
PPY-A	0.83	27.78	50	61.11	0.81	21	47	50
SX7	0.91	26.32	60.53	76.32	0.91	42	52	66
DCC-2036	0.82	45.95	45.95	54.05	0.91	19	52	64
Ponatinib	0.85	47.22	55.56	61.11	0.92	50	56	71

AUC, area under the curve; EF, enrichment factor; MM-GBSA, molecular mechanics – generalized Born surface area; ROC, receiver operating characteristic; SP, standard precision.

#### VS with Glide decoys and weak inhibitors of ABL1

As it was most successful, the ponatinib-bound ABL1-T315I conformation was chosen for further VS studies to test the effects of alternate choices for decoys and alternate methods for binding energy calculations. Using either the ‘universal’ Glide decoys or ABL1 weak binders as decoy sets, ranked hit lists from SP and/or XP docking runs were either used directly or re-ranked using the MM-GBSA approach with a rigid receptor model or using the MM-GBSA approach with receptor flexibility within 12 Å of the ligand. Table6 summarizes the results. For the Glide decoys, SP docking was sufficient to eliminate 86% of decoys, partially at the cost of low early enrichment values, which MM-GBSA energy calculations were not able to improve. The ABL1 weak inhibitor set was used as the strongest challenge to VS runs, because these, as ABL1 binders, require highest accuracy in binding energy ranking for recognition. And indeed, SP docking eliminated only roughly 50%, in contrast to the results for the Glide ‘universal’ decoys. However, the XP docking was able to improve this to eliminate some 83%, at the cost, however, of eliminating a larger set of active compounds. Both ROC and early enrichment values show that XP docking performed better than random for the reduced set of compounds classified as hits, but only barely. The addition of MM-GBSA calculations with the rigid and flexible receptors did not offer significant improvement.

**Table 6:** Virtual screening (VS) with glide decoys and weak inhibitors of ABL1. The ponatinib-bound ABL1-315I conformation was used for VS runs

Ligand of target kinase	Scoring function	Decoys identified as hits (%)	ROC AUC	EF1%	EF5%	EF10%
Glide decoys	SP	14.4	0.99	3	24	50
	SP:MM-GBSA		0.96	3	24	50
	SP:MM-GBSA12		0.92	3	24	47
ABL1 weak inhibitors (100–1000 nm)	SP	42.36	0.65	3	9	12
	SP:MM-GBSA		0.70	3	9	12
	SP:MM-GBSA12		0.59	0	9	9
	XP	17.24	0.58	0	0	5
	XP:MM-GBSA		0.64	5	10	20
	XP:MM-GBSA12		0.63	0	0	15

AUC, area under the curve; EF, enrichment factor; MM-GBSA, molecular mechanics – generalized Born surface; ROC, receiver operating characteristic; SP, standard precision; XP, extra precision.

### Ligand-based studies

#### Chemical space of active inhibitors

**Figure 6:**
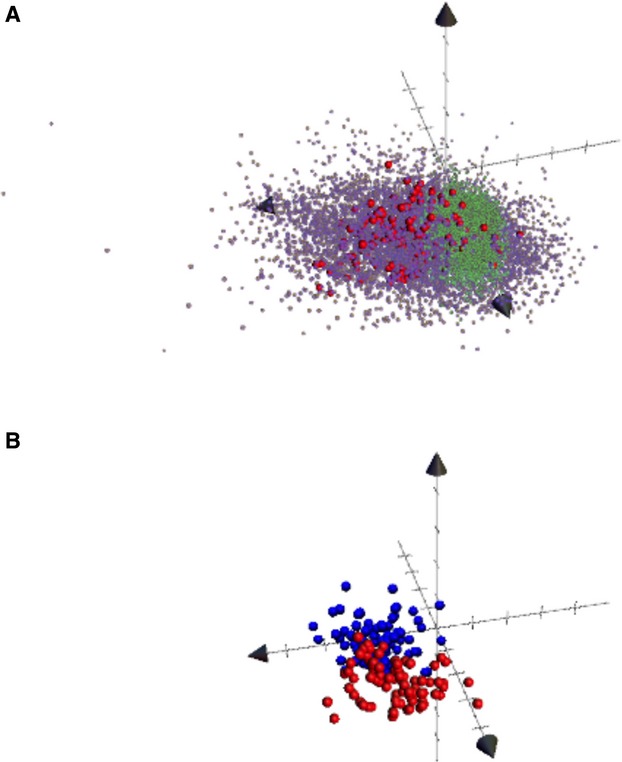
Chemical spaces occupied by active inhibitor and decoys. About 40 molecular properties were summarized to eight principal components (PCs), and three major PCs were mapped in three-axes of Cartesian coordinates. (A) Color coded as blue is for randomly selected potent kinase inhibitors, green is for Directory of Useful Decoys (DUD) decoys, and red is for highly potent dual activity ABL1 inhibitors. (B) Blue is for ABL1-wt and red for ABL1-T315I. PC1, which is predominantly size, shape, and polarizability, distinguishes DUD decoys and inhibitors most.

Despite some overlap, active inhibitors and DUD decoys map to distinguishable volumes in chemical space (Figure6A). This itself provides information to filter sets of potential inhibitors to eliminate compounds that match decoys rather than inhibitors. In contrast, plotting ABL1-wt selective inhibitors versus dual active ABL1 inhibitors does not distinguish the sets (Figure6B) in the major PC dimensions.

#### Correlation of molecular properties and binding affinity

Multiple calculations were made to identify the strongest linear correlations between the molecular properties of the inhibitors and their experimental pIC_50_ values. For ABL1-wt, the numbers of hydrogen bond donors and rotatable bonds showed the strongest correlations (*R*^2^ of 0.87 and −0.69, respectively). In contrast, for ABL1-T315I, only the number of rotatable bonds showed a strong correlation (*R*^2^ = −0.59), consistent with loss of threonine as a hydrogen bonding acceptor in the ABL1-T315I mutant. In both cases, the number of rotatable bonds was found to negatively correlate with the pIC_50_ values with moderate correlation, supporting the generally valid inhibitor design goal that minimizing flexibility will enhance binding (provided the ability to fit the binding site is maintained, of course).

**Figure 7:**
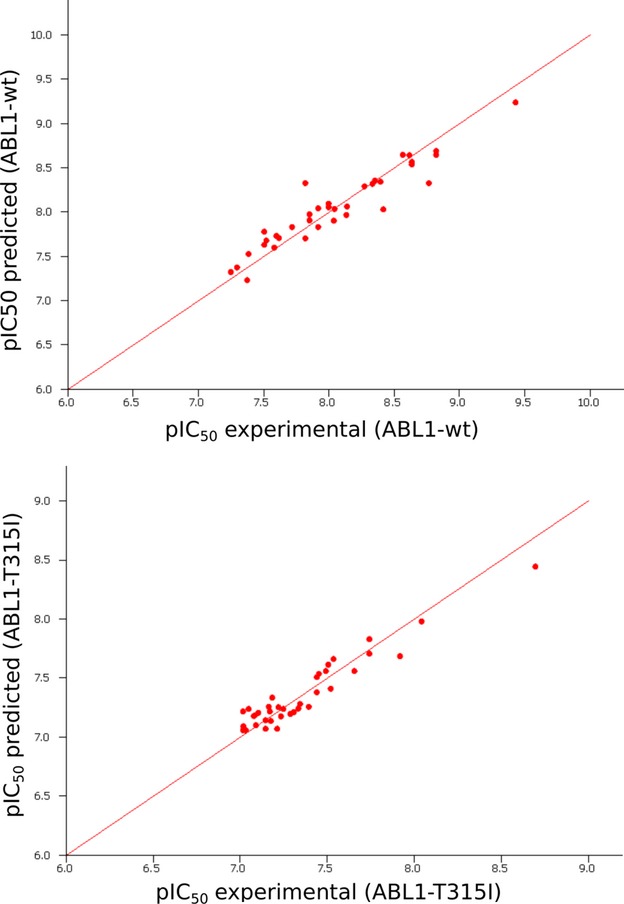
Neural network–based prediction of pIC_50_ values of the active inhibitors from their molecular properties.

Several methods (multiple linear regression, PLS regression, and neural network regression) were used to create models for predicting the experimental binding affinity (pIC_50_) from molecular properties. Even in the absence of clear correlations with individual molecular properties, such models can in principle be trained to recognize complex multifactorial patterns, given enough data. Here, the neural network–based regression provided the best correlation between the experimental and predicted values (Figure7).

## Discussion

### Structure-based studies

#### ABL1 kinase domain structure

**Figure 8:**
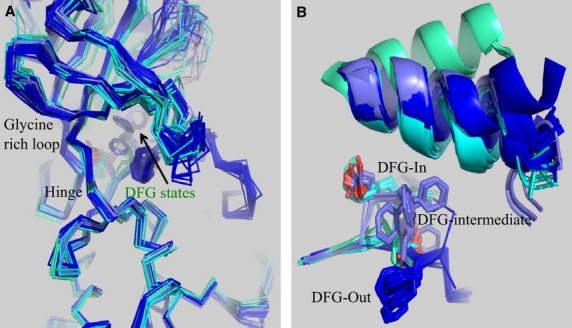
Overview of published ABL structures showing the clustering of DFG states at the ATP binding pocket. (A) The location of the DFG conformation clusters relative to the hinge (front). The positions of the DFG phenylalanine affect the ATP pocket volume most significantly and cluster into several groups. (B) Detail of the clustering of DFG states including the positions of the C helix: DFG-in (cyan), DFG-out (dark blue), inactive DFG-intermediate (steel blue), and DFG-'src like’ (turquoise), the latter represented by a single Protein Databank (PDB) entry (2g1t).

Some 40 crystal structures of ABL kinase domains (including point mutants and ABL2) are available in the Protein Databank (PDB), providing a good picture of the plasticity of the receptor. Key variations are seen in the positions of the activation and the glycine-rich loops, which are of a scale too large for automated receptor flexibility algorithms to have a chance of correct prediction. However, they do cluster into clearly distinct groups (Figure8), and representatives of the groups may be selected for use in drug discovery tasks. The extent of knowledge of drug target plasticity depends on extensive crystallography research, something not available for relatively new targets. On the other hand, for key target classes, such as protein kinases, it is quickly becoming the norm to have significant information regarding structural plasticity of the target in drug discovery programs.

By itself, knowledge of target plasticity is not sufficient for good predictivity of inhibitor binding properties. For example, the energy costs of reorganization must be taken into account, and these are not generally accessible to theoretical methods. Instead, one increasingly has recourse to databases of ligand binding energies. As these databases grow, the prediction of binding energies from known binding data and explicit consideration of the plasticity of target structures will improve. At some point, the size and diversity of the binding data alone may become sufficient for predictivity when used in ‘high-data-volume’ 3D-QSAR-type approaches. At present, as can be seen here and elsewhere in the literature, ligand-alone data are not adequate for binding predictivity, outside of narrowly proscribed boundaries, and drug design methods benefit greatly from consideration of target structures explicitly.

For tyrosine kinases, notably including ABL, the distinction between ‘DFG-in’ and ‘DGF-out’ states arises from the conformation of the activation loop and generates the major classification of inhibitor types (I and II, respectively) Among the type I conformations, substantial variations can be found, especially concerning the glycine-rich loop and the conformation of the DFG motif, such that the classification becomes less clear. For example, the SX7 structure shows the DFG motif to occupy a conformation intermediate between ‘DFG-in’ and ‘DGF-out’ (Figure7). Also, the danusertib-bound structure (PDB: 2v7a) shows the glycine-rich loop in an extended conformation, whereas the other eight structures show the loop in a shared bent conformation in close contact with inhibitors.

The ‘DFG-in’ conformation corresponds to the active state of the kinase, whereby the loop is extended and open, the phenylalanine residue of DFG occupies a hydrophobic-aromat binding site at the core of the kinase domain, and the aspartic acid is poised to coordinate a magnesium ion which in turn coordinates the beta and gamma phosphate groups of ATP. In the DFG-in conformation, the kinase domain can bind both ATP and protein substrate, and the adenine ring of the ATP can form hydrogen bonds to the hinge region of the kinase domain 24. In contrast, the ‘DFG-out’ conformation represents an inactive form of the kinase (Figure1C) and is generally incompatible with both nucleotide and protein substrate binding. This conformation was first seen in an ABL1 complex with imatinib 25, but has since been found for many inhibitors and many kinases. In this conformation, the DFG segment is rotated, removing the DFG aromat from its binding site and creating a cavity, which can tightly accommodate inhibitors. The phenylalanine side chain can also partially occlude the ATP binding pocket. ABL inhibitor complex structures in the PDB show both DFG-in and DFG-out conformations, for both wild-type and T315I forms, as described above. Type II inhibitors (DFG-out binders) block the conformational change to the DFG-in state and so bind only to the DFG-out conformation. Type I inhibitors may bind both DFG-in and DFG-out conformations. These two conformations do not define two distinct and rigid states for the protein, and a new pharmacophore type I 1/2 has been proposed recently, which includes inhibitors with all type I but few type II interactions 26. Although no inhibition data are publically available for SX7, the KKB shows a few congeners of SX7 that weakly inhibit FLT1, FGFR1, and Aurora kinase A. But four other ligands are highly potent inhibitors of both forms of ABL1 kinase. Therefore, the nine publicly available PDB structures form complete representative set for a thorough VS study. A similar study has been published recently that used several crystal structures of p38γ to investigate the effects of combining hit lists from different crystal structures of the same target 27.

#### Ligand selection

The choice of target structures and the choice of ligands were guided by the aim to represent target plasticity on the one hand and to preserve desirable high-potency dual inhibition qualities on the other hand. The decoy sets were chosen to enable testing of the VS methods in general, but also to test the applicability of different types of decoy sets. The DUD set was chosen to best match ABL1 inhibitor-like ligands, and the Glide set is a universal decoy set. The DUD decoy set has been previously used for enrichment studies 28. The DUD data set has been shown to include analog bias in the active ligand set. However, a recent version of DUD data set (DUD-E) has been released to address analog bias by enhancing chemotype diversity by their Bemis–Murcko atomic frameworks 29. Alternate data sets are available that also aim to minimize analog bias, such as Maximum Unbiased Validation (MUV) 30. In this study, we have replaced the original active ligands from the DUD data set with the inhibitors retrieved from KKB, so the analog bias of the DUD active ligands is not present.

One interesting result was the differentiation between the type II receptor conformations, namely 3ik3 (ponatinib bound) and 3qrj (DCC-2036 bound). With SP docking, about 30% of DUD decoys were predicted as hits, whereas this was more than 50% for 3qrj. The early enrichment (EF1%) was also different between these conformations: 47.37% for 3ik3 and 61.11% for 3qrj. The enrichment is similar for EF5%. Thus, the type II conformation represented by the ponatinib-bound ABL1-T315I structure performed better for enriching active inhibitors; the large proportion of ponatinib like inhibitors in the dual active set probably accounts for this.

Directory of Useful Decoys decoy set has been previously used for enrichment studies 28. Using the Glide universal decoys, only 14.4% of decoys were predicted as hits. This is an encouraging indicator, especially during VS with unfocussed ligand library. The early enrichment values between DUD and Glide decoys are not easily comparable, however, because of the different total content of decoys in the hit sets – inclusion of only few decoys in the hit list dramatically reduces the EF values. Therefore, low early enrichment values with a small decoy set (such as Glide decoys here) should be a discouraging indicator in VS.

Using weak ABL1 binders as the decoy set – the most challenging variety – the Glide XP method was remarkably able to eliminate some 80% of the decoys, whereas the SP method eliminated about 60%. After elimination, the overall enrichment (indicated by ROC AUC) values were similar.

#### Chemical space navigation

ABL active inhibitors seem underrepresented in the KKB data set. More than 23000 ligands are recorded with IC_50_ < 100 nm for all forms of kinases, but only 255 are active against ABL1 (wild-type and mutant forms). This has been shown in a recent study with more than 20 000 compounds against a 402-kinase panel 31. Of the 182 dual activity inhibitors, 38 showed high activity (IC_50_ < 100 nm) for both the receptor forms. But 90 high-activity ABL1-wt receptor showed medium (IC_50_ = 100–299 nm) or low (IC_50_ = 300–1000 nm) activity for ABL1-T315I. A few inhibitors – less than 10 – showed high activity for ABL1-T315I, but medium to low activity for ABL1-wt.

## Conclusion

In this study, VS methods were applied to test their ability to identify inhibitors of leukemia target kinase ABL1 and its drug-resistant mutant form T315I. Nine PDB structures of the ABL1 kinase domain, with and without the mutation, and representing different activation forms, were used for GLIDE docking. ABL1 inhibitors were retrieved from Kinase Knowledge Base (KKB) database and combined with decoy compounds from the DUD database. Enrichment factor and receiver operating characteristic (ROC) values calculated from the VS studies show the importance of selecting appropriate receptor structure(s) during VS, especially to achieve early enrichment.

In addition to the VS studies, chemical descriptors of the inhibitors were used to test the predictivity of activity and to explore the ability to distinguish different sets of compounds by their distributions in chemical space. We show that VS and ligand-based studies are complementary in understanding the features that should be considered during *in silico* studies.
